# Bite force of patients with tooth pain

**DOI:** 10.1002/cre2.565

**Published:** 2022-07-15

**Authors:** Yoshinobu Shoji, Mohd Yusmiaidil Putera Mohd Yusof, Rostam Iffendi Bin Idris, Somsak Mitrirattanakul

**Affiliations:** ^1^ Centre for Oral & Maxillofacial Diagnostics and Medicine Studies, Faculty of Dentistry Universiti Teknologi MARA Selangor Sungai Buloh Selangor Malaysia; ^2^ Laboratory and Forensic Medicine (I‐PPerForM), Institute of Pathology Universiti Teknologi MARA Selangor Sungai Buloh Selangor Malaysia; ^3^ Centre for Restorative Dentistry, Faculty of Dentistry Universiti Teknologi MARA Selangor Sungai Buloh Selangor Malaysia; ^4^ Department of Masticatory Science, Faculty of Dentistry Mahidol University Bangkok Thailand

**Keywords:** bite force, prescale film, tooth pain

## Abstract

**Objectives:**

The aim of this study was twofold: (i) to measure the bite force of healthy adults and patients with tooth pain and (ii) to evaluate the influence of bite force and age on tooth pain and both genders. It is hypothesized that patients with tooth pain would have lesser bite forces as compared to healthy individuals.

**Material and Methods:**

Two groups of participants were, the first group comprised 18 healthy adults (9 males, 9 females), while the second group comprised 18 patients with tooth pain (9 males, 9 females), recruited from the Faculty of Dentistry, Universiti Teknologi MARA. Their maximum bite forces were recorded using the Prescale system that consists of pressure‐sensitive films and a precalibrated scanning device. Logistic regression models were used using bite force and age on dichotomous responses of tooth pain status and gender.

**Results:**

The mean bite force of patients with tooth pain was 684.77 ± 501.13 N, which was lesser than 798.33 ± 492.16 N of the healthy adults. The reduced gender logistic regression model on gender with age was found to be statistically significant (*p* ≤ .05).

**Conclusions:**

Even though the mean bite force was smaller in the group with dental pain, this difference was not statistically significant.

## WHY THIS PAPER IS IMPORTANT

- This study was designed to measure the bite forces of patients with clinical tooth pain and healthy individuals. It is hypothesized that patients with tooth pain would have lesser bite forces as compared to healthy individuals.

## 1 INTRODUCTION

Bite force is one of the indicators of the functional state of the masticatory system that results from the action of jaw elevator muscles modified by the craniomandibular biomechanics (Bakke, 2006). The measurement of the bite forces can yield important and useful information for the evaluation of jaw muscle function and activity.

Some factors that affect bite force measurements have been reported, such as craniofacial morphology, age, gender, periodontal support of teeth, signs, and symptoms of temporomandibular disorders (TMD) and pain, as well as dental status (Koc et al., 2010). For an improved understanding of the correlation between biting activity and induced dental pain, several animal models have been developed. Foong et al. (1982) found measurable changes in jaw movement patterns during biting and Sunakawa et al. (1999) found increased electrical activity in the masseter muscle, suggesting a functional link between dental pain and masticatory function (Benoliel et al., 2002; Khan et al., 2008). A study by Awawdeh et al. (2017) found that endodontically treated teeth have higher maximum bite force compared to the control group due to the role of the dental pulp as a sensitive sensor.

However, the role of clinical tooth pain on bite force has not been explored. The aim of this study was twofold: (i) to measure the bite force of healthy adults and patients with tooth pain and (ii) to evaluate the influence of bite force and age on tooth pain in both males and females. It is hypothesized that the bite force of patients with tooth pain would be lesser than those of healthy adults.

## 2 MATERIALS AND METHODS

### 2.1 Recruitment and selection of subjects

An oral examination was performed to verify that participants fulfilled the study inclusion criterion, that is, the presence of at least 20 natural teeth in the mouth. A thorough extra and intraoral examination was performed to verify that participants fulfilled the study inclusion criterion, that is, the presence of at least 20 natural teeth in the mouth. For extraoral examination, inspection and palpation were conducted by the operator on patients’ facial and neck regions. Any abnormalities like swelling, infection, or wound were noted. For intraoral examination, patients were lying supine on the dental chair and consent for oral examination was gained verbally. The oral inspection was performed using a mouth mirror and the obvious periodontal problem was checked using a periodontal probe.

Thirty‐six participants were recruited for this study. The first group consisted of 18 eligible participants who were all students with ages ranging from 19 to 23 years (mean age of 21 years; 9 males, 9 females). They were recruited from the Faculty of Dentistry, Universiti Teknologi MARA (UiTM) to serve as healthy controls. All these participants met the eligibility criteria for the study, which were asymptomatic from the pain of dental origin. Exclusion criteria for the asymptomatic participants were subjects with systemic diseases that affect jaw functions, developmental anomalies, TMDs, periodontal disease, or any history of jaw injury, trauma, or neoplastic disease. Participants who were undergoing orthodontic treatment were also excluded.

The second group involved 18 consecutive patients who were all adults with ages ranging from 19 to 36 years (mean age of 25 years; 9 males, 9 females). Exclusion criteria for the patients were the same as the asymptomatic participants. All cases were recruited through the UiTM dental centre and diagnosed by the orofacial pain specialist who made a clinical judgment that the pain was of dental origin. Participants who need to have immediate treatment for their pain were excluded. The pain intensity was not evaluated.

This study was approved by the Research Ethics Committee of UiTM (600‐IRMI, REC/108/17), and all subjects were provided written informed consent as part of the study protocol.

### 2.2 Bite force measurement and analysis

Their maximum bite forces were recorded using the Prescale system that consists of pressure‐sensitive films (Dental Prescale®, Type‐R 50H; Fuji Film, Tokyo, Japan) and a precalibrated scanning device (Occluzer® FPD‐707; GC Co., Tokyo, Japan). All subjects were asked to bite on the film for 3 s with maximal bite force at their intercuspal positions. Before the measurement, subjects were seated comfortably on a dental chair in a natural unsupported posture looking forward, to the greatest extent possible, with the Frankfort horizontal plane parallel to the floor. The subjects were then asked to occlude repetitively in an intercuspal position to ensure that the teeth were in maximal contact when they bit on the pressure‐sensitive film, which is a horseshoe‐shaped disposable sheet (thickness, 0.097 mm). All subjects were then asked to bite three times on the films for 3 s with a maximum bite force. There was a time interval of 5 min in each biting. The average bite force was calculated from the two readings obtained.

The procedures were repeated three times where the mean bite forces of each subject were calculated and recorded. The system has been well documented by Matsui et al. (1996) and Chong et al. (2016).

### 2.3 Statistical analysis

Data were analysed using RStudio, PBC version 0.97.551‐©2009–2012 Inc. software,. Mean bite force was compared between the two groups by using the *t* test (*p* < .05). Two logistic regression models were performed using bite force and age as continuous predictor variables, respectively. The first model used gender, while the second model utilized the presence of tooth pain as binary responses. A likelihood ratio test (LRT) was used to compare nested models. This test yields a *χ* ^2 ^value and a *p *value. If two nested models are compared and the LRT produces *p* ≤ .05, then the model being compared fits the data better than the base model. The gender and presence of tooth pain were dichotomized into 0 = female and 1 = male and 0 = no pain and 1 = pain, respectively, for both models.

## 3 RESULTS

### 3.1 Bite force measurements

**Figure 1:**
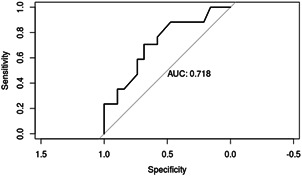
The area under the receiver‐operating characteristic curve for the reduced model (gender–age)

The bite force of healthy adults ranged from 86.40 to 1758.60 N; the mean (SD) bite force was 798.33 ± 492.16 N. The bite forces of the patients with tooth pain ranged from 119.0 to 1648.40 N, while the mean bite force (SD) was 684.77 ± 501.13 N (Table 1). The calculated mean bite forces of patients with tooth pain resulted in a smaller value as compared to healthy adults. However, the difference was not significant. The relationship between occlusal force and gender is shown in Figure 1. Although the mean occlusal force of males is higher than that of females, there was no significant difference in bite force between genders among both patients with tooth pain and healthy adults (*p* = .201). Also, there was no significant difference in bite force of each group according to gender (male, *p* = .389; female *p* = .389).

**Table 1:** Details of patients with pain of dental origin

Age range (years)	Gender	Diagnoses	Mean bite force (N)	SD
19–36	Female (*n* = 9)	Periodontitis, irreversible pulpitis, pericoronitis, apical periodontitis, reversible pulpitis, periapical abscess, periapical infection, hypersensitivity	648.77	501.13
	Male (*n* = 9)			

Abbreviation: SD, standard deviation.

The reduced gender logistic regression model (gender–age) was found to be statistically significant with a coefficient estimate of .05 (*p* ≤ .05) as compared to all other full models and reduced gender/pain logistic regression models. The percentage of gender–age model's area under the curve is 72% (Figure 1).

## 4 DISCUSSION

In this study, the bite force of patients with pain of dental origin was smaller than those healthy adults, but it was not statistically significant.

The maximum voluntary bite force closely correlates with age, gender, muscle condition, and the number of teeth, and also with factors such as the measuring apparatus used (Helkimo et al., 1977; Ingervall & Helkimo, 1978). The occlusal force is known to be age‐related. Several studies have shown that the occlusal force tends to increase through various stages of development, but stabilizes after puberty. They reach their peak at 12 years of age, stabilize after the age of 14 years, and decline slightly by the age of 17 years (Braun et al., 1996; Shinogaya et al., 1999). In the present study, the inclusion criteria are that the patients must be aged 18 years and above to reduce sample bias.

Gender is also known to be another factor that affects the occlusal force. In general, present results showed that the bite force decreases significantly with aging women as compared to aging men (*p* ≤ .05). Previous studies concurred with the present finding. Bakke et al. (1990) have reported that bite force decreases with age after 25 years in females and after 45 years in males. Bite force decreases significantly with age, especially in women. Shinogaya et al. (2000, 2001) have evaluated the effects of age on maximum bite force, average magnitudes of pressure, and occlusal contact areas in elderly (53–62 years) and young (20–26 years) Japanese subjects.

Subsequently, males possess a higher mean occlusal force than the females in both groups, although the differences were not statistically significant. This result is consistent with the study carried out by Dean et al. (1992), in which the occlusal force was correlated with masticatory muscle thickness and gender. This is attributed to the excretion of ketosteroids in postpubertal young men, which increases the muscle mass (Tanner et al., 1959). Therefore, greater muscle mass development in males could result in gender‐related occlusal force differences in the postpubertal population. The findings obtained in the present study are in accordance with the previous literature.

Bite force during chewing is not only correlated with maximal strength of the masticatory muscles but is also probably controlled by other factors such as pain thresholds (Mohl et al., 1988). Generally, patients with irreversible pulpitis may have smaller bite force as compared to the other conditions. The reason is that the spread of pulp infection to the periodontal tissues and periodontal ligament stimulates the periodontal nociceptors to transmit mainly fast pain, and thus it plays a role in rapid detection of the injury‐related stimuli during mastication and may induce the masseteric inhibitory reflex, leading to a jaw opening reflex (McGrath et al., 1981). Toda et al. (2004) reported that most nociceptors of rat's periodontal nociceptors were A delta‐innervated, while only a small number of C‐innervated nociceptors were found. The results suggest that periodontal nociceptors transmit mainly fast pain, and may thus play a role in the rapid detection of injury‐related stimuli during mastication. Participants who need to have immediate treatment for their pain were excluded in this study; therefore, the influence of A delta‐innervated nociceptors may be reduced.

In the present study, all participants had 20 or more teeth, including their posterior teeth. Arnold (1981) reported that the molars, premolars, and incisors have a bite force ratio of 4:2:1, that is, the forces are much greater on the posteriormost teeth, which are close to the muscles producing the force.

There are several occlusal force measurement devices available in the market such as the Photocclusion® and T‐scan system®. The photocclusion system manages to record the color patterns of the occlusal contact, but quantitative measurement of this device was difficult and the sheet was rigid (Amsterdam et al., 1987). On the other hand, the T‐scan system® measured occlusal force based on changes in electrical resistance. This system offered only a narrow range of measurement for the occlusal force, and due to different sensitivity, it tended to record fewer occlusal contacts than were present as checked by occlusal foils (Suzuki et al., 1997). Other than that, the thick sensor may affect the function of the masticatory muscles. Dental Prescale/Occluzer® has been studied in many aspects of dentistry. This system enables accurate measurements for a wide range of pressure (5–120 MPa) and the thin material induces only a small change in the occlusal vertical dimension, making measurements at a position near the intercuspal position possible in the mouth. Also, it does not require a hand‐held device with a sensor, many patients can be evaluated in a short period, and record storage is simplified even for an extended period.

There was a limitation in this study. The pain intensity and psychological status that would be able to influence the bite force were not evaluated. The future study could have these evaluations as well.

## 5 CONCLUSION

From the results, even though the mean bite force was smaller in the group with dental pain, this difference was not statistically significant.

## AUTHOR CONTRIBUTIONS

Yoshinobu Shoji conceived and planned the experiments. Yoshinobu Shoji and Rostam Iffendi Bin Idris carried out the experiments. Yusmiaidil Putera Mohd Yusof contributed to the interpretation of the results and helped shape the analysis. Somsak Mitrirattanakul supervised the findings of this study. All authors discussed the results and provided critical feedback to the final manuscript.

## CONFLICTS OF INTEREST

The authors declare no conflicts of interest.

## Data Availability

The data that support the findings of this study are available on request from the corresponding author. Data are openly available in a public repository that issues data sets with DOIs.
